# Supplementary material for: Inferring interaction partners from protein sequences using mutual information
Source: PLoS Comput Biol. 2018 Nov 13;14(11):e1006401. doi: 10.1371/journal.pcbi.1006401 (PMC6258550; doi:10.1371/journal.pcbi.1006401)
Supplement: S1 Table — MI and DCA scores trained on the full alignment of cognate HK-RR pairs (excluding orphans, see Methods) were used to score all possible HK-RR pairs, including orphans, in species where orphan and crosstalk partners have been reported in the literature. For each HK considered, RRs were ranked using the MI and DCA scores. The table indicates the species, the HK and RR names, the literature references where partnership was reported, the total number of RRs in the species considered, and the rank among all these RRs of the RR considered, both using MI scores and using DCA scores. Finally, we report a comparison of the DCA ranking with the previous one in [14]: does our ranking give a better (higher) or a worse (lower) rank to the known partner than this previous ranking? Special mentions in the table: (pair): the cognate pair was included, in order to compare it with the crosstalk partner; Worse*: 2 ranks lower (including an inversion between PleD and DivK, which had close scores in [14]); Worse**: 1 rank lower (including an inversion between PleD and DivK, which had close scores in [14]). (PDF) [file pcbi.1006401.s008.pdf]

S1 Table for *Inferring interaction partners from protein sequences using mutual information* by Anne-Florence Bitbol

Species	HK usual name	HK ordered locus name	RR usual name	RR ordered locus name	Ref.	Number of RRs	MI rank	DCA rank	Comparison with [1]
Thermotoga maritima		TM0853		TM0468	[2]	11	2	2	
Synechococcus elongatus	SasA/CikA	Synpcc7942_2114	SrrA	Synpcc7942_2416	[3]	21	2	2	
Synechococcus elongatus	SasA/CikA	Synpcc7942_2114	RpaB	Synpcc7942_1453	[3]	21	6	4	
Synechococcus elongatus	SasA/CikA	Synpcc7942_2114	RpaA	Synpcc7942_0095	[3, 4]	21	7	5	
Synechococcus elongatus		Synpcc7942_0453	Ycf29	Synpcc7942_1860	[3]	21	2	12	
Synechococcus elongatus		Synpcc7942_2242	SrrB	Synpcc7942_0556	[3]	21	8	5	
Synechococcus elongatus	NblS	Synpcc7942_0924	RpaB	Synpcc7942_1453	[3]	21	1	1	
Synechococcus elongatus	NblS	Synpcc7942_0924	SrrA	Synpcc7942_2416	[3]	21	3	4	
Synechocystis	Hik2	slr1147	Rre1	slr1783	[3, 5]	41	2	7	
Myxococcus xanthus	CrdS	MXAN_5184	CrdA	MXAN_5153	[6]	138	1	1	
Myxococcus xanthus	SasS	MXAN_1249	SasR	MXAN_1245	[6]	138	9	3	
Caulobacter crescentus	DivJ	CC_1063	DivK	CC_2463	[7]	46	8	1	Same
Caulobacter crescentus	DivJ	CC_1063	PleD	CC_2462	[7]	46	13	2	Same
Caulobacter crescentus	PleC	CC_2482	PleD	CC_2462	[7]	46	2	1	Better
Caulobacter crescentus	PleC	CC_2482	DivK	CC_2463	[7]	46	3	4	Worse*
Caulobacter crescentus	CenK	CC_0530	CenR	CC_3743	[1, 7]	46	17	7	Better
Caulobacter crescentus	DivL	CC_3484	DivK	CC_2463	[1]	46	16	24	Worse
Caulobacter crescentus		CC_1062	DivK	CC_2463	[1]	46	14	2	Worse**
Clostridium acetobutylicum		CA_C0903	Spo0A	CA_C2071	[8]	42	22	12	
Clostridium acetobutylicum		CA_C3319	Spo0A	CA_C2071	[8]	42	22	12	
Bacillus subtilis	KinA	BSU13990	Spo0F	BSU37130	[1]	35	1	1	Same
Bacillus subtilis	KinB	BSU31450	Spo0F	BSU37130	[1]	35	6	3	Better
Bacillus subtilis	KinC	BSU14490	Spo0F	BSU37130	[1]	35	6	1	Same
Bacillus subtilis	KinD	BSU13660	Spo0F	BSU37130	[1]	35	1	1	Same
Bacillus subtilis	KinE	BSU13530	Spo0F	BSU37130	[1]	35	2	1	Same
Streptococcus mutans (pair)	VicK	Smu_1516	VicR	Smu_1517	[9]	15	1	1	
Streptococcus mutans	VicK	Smu_1516	GcrR	Smu_1924	[9]	15	2	2	

Orphan partnership and crosstalk prediction in HK-RRs by MI and DCA. MI and DCA scores trained on the full alignment of cognate HK-RR pairs (excluding orphans, see Methods) were used to score all possible HK-RR pairs, including orphans, in species where orphan and crosstalk partners have been reported in the literature. For each HK considered, RRs were ranked using the MI and DCA scores. The table indicates the species, the HK and RR names, the literature references where partnership was reported, the total number of RRs in the species considered, and the rank among all these RRs of the RR considered, both using MI scores and using DCA scores. Finally, we report a comparison of the DCA ranking with the previous one in [1]: does our ranking give a better (higher) or a worse (lower) rank to the known partner than this previous ranking? Special mentions in the table: (pair): the cognate pair was included, in order to compare it with the crosstalk partner; Worse*: 2 ranks lower (including an inversion between PleD and DivK, which had close scores in [1]); Worse**: 1 rank lower (including an inversion between PleD and DivK, which had close scores in [1]).

References cited in S1 Table

1. A. Procaccini, B. Lunt, H. Szurmant, T. Hwa, and M. Weigt. Dissecting the specificity of protein-protein interaction in bacterial two-component signaling: orphans and crosstalks. *PLoS ONE*, 6(5):e19729, 2011. **Cited as reference 14 in the main text.**

2. P. Casino, A. Fernandez-Alvarez, C. Alfonso, G. Rivas, and A. Marina. Identification of a novel two component system in Thermotoga maritima. Complex stoichiometry and crystallization. *Biochim. Biophys. Acta*, 1774(5):603–609, May 2007.

3. H. Kato, S. Watanabe, K. Nimura-Matsune, T. Chibazakura, Y. Tozawa, and H. Yoshikawa. Exploration of a possible partnership among orphan two-component system proteins in cyanobacterium Synechococcus elongatus PCC 7942. *Biosci. Biotechnol. Biochem.*, 76(8):1484–1491, 2012.

4. A. Gutu and E. K. O'Shea. Two antagonistic clock-regulated histidine kinases time the activation of circadian gene expression. *Mol. Cell*, 50(2):288–294, Apr 2013.

5. R. Vidal. Identification of the correct form of the mis-annotated response regulator Rre1 from the cyanobacterium Synechocystis sp. PCC 6803. *FEMS Microbiol. Lett.*, 362(7), Apr 2015.

6. J. W. Willett, N. Tiwari, S. Muller, K. R. Hummels, J. C. Houtman, E. J. Fuentes, and J. R. Kirby. Specificity residues determine binding affinity for two-component signal transduction systems. *MBio*, 4(6):e00420–00413, Nov 2013.

7. J. M. Skerker, M. S. Prasol, B. S. Perchuk, E. G. Biondi, and M. T. Laub. Two-component signal transduction pathways regulating growth and cell cycle progression in a bacterium: a system-level analysis. *PLoS Biol.*, 3(10):e334, Oct 2005.

8. E. Steiner, A. E. Dago, D. I. Young, J. T. Heap, N. P. Minton, J. A. Hoch, and M. Young. Multiple orphan histidine kinases interact directly with Spo0A to control the initiation of endospore formation in Clostridium acetobutylicum. *Mol. Microbiol.*, 80(3):641–654, May 2011.

9. J. S. Downey, L. Mashburn-Warren, E. A. Ayala, D. B. Senadheera, W. K. Hendrickson, L. W. McCall, J. G. Sweet, D. G. Cvitkovitch, G. A. Spatafora, and S. D. Goodman. In vitro manganese-dependent cross-talk between Streptococcus mutans VicK and GcrR: implications for overlapping stress response pathways. *PLoS ONE*, 9(12):e115975, 2014.
